# 

**Published:** 1900-07-07

**Authors:** 


**DEATH.**

June 23rd, 1900, at Malvern, MARGARET ELIZABETH HENNIKER, aged 27, late of Victoria Hospital, Chelsea, after a year of intense suffering. (277)

### NOTICE TO CORRESPONDENTS.

All MS., Letters, Books for Review, and other matters intended for the Editor should be addressed The Editor, "The Hospital," The Hospital Building, 28 & 29, Southampton Street, Strand, W.C.

The Editor cannot undertake to return rejected MS., even when accompanied by stamped directed envelope.

**Notice to Advertisers and Subscribers.**

All Advertisements, Orders for Copies of The Hospital, and Business Communications should be addressed to **THE MANAGER (Not to the Editor)**, The Hospital, The Hospital Building, 28 & 29, Southampton Street, Strand, London, W.C.

The Cost of Subscription, post free, is as follows:—Per week, 2½d.; per quarter, 2s. 8d.; per half-year, 5s. 3d.; per annum, 10s. 6d. Foreign:—Per annum, 15s. 2d., payable, in all cases, in advance.

**NOTICE.—Vol. XXVII. of "The Hospital" (October, 1899, to April, 1900), in handsome cloth binding, is now on sale at the Office of this Journal, price 7s. 6d. Cases for binding the Numbers contained in this Volume 1s. 6d. Index to Vol. XXVII., 2½d., post free.**

**The Monthly Part for July is now ready. Price 6d., by post 8d.**

### The Student of Medicine.

### APPOINTMENTS.

H. J. Davis, M.A., M.B.Cantab, M.R.C.P.Lond., appointed Assistant Physician to the West London Hospital. A. Dey, M.B., C.M.Aberd., appointed Medical Officer of Health to the Glendale Rural District. J. T. Farrell, L.R.C.S. and L.R.C.P.Edin., appointed Dispensary Medical Officer and Medical Officer of Health for Dungloe No. 1 Dispensary District of the Glenties Union. H. J. Flanegan, L.R C.S., L.R.C.P.Irel., appointed Medical Officer for the All Saints' District of the Northampton Union. P. Foster, M.R.C.S. Eng., L.R.C.P.Lond., appointed House Physician to the Sussex County Hospital, Brighton. F. Hollinshead, M.D.St. And., appointed Visiting Medical Officer to the King's Norton Union Infirmary. J. Hora, M.B., B.S.Lond., appointed Resident Medical Officer to the King's Norton Union Infirmary. E. B. Hulbert, M.D.Durh., M.R.C.S., L.R.C.P.Lond., appointed Second Assistant Medical Officer to the St. Marylebone Infirmary. J. M. Longford, L.R.C.P., L.R.C.S.Irel., appointed Assistant Medical Officer to the Cottage Homes of the Aston Union. C. D. Marshall, F.R.C.S., appointed Assistant Surgeon to the Royal London Ophthalmic Hospital (Moorfields Eye Hospital), City Road, E.C. A. M. Mitchell, M.A., M.D., B.C.Cantab., D.P.H.Camb., appointed Medical Officer of Health for the Borough of Guildford. J. P. Race, L.S.A., appointed Assistant Medical Officer to the Gordon Road Workhouse, Camberwell. J. A. Robertson, M.D. Glasg., appointed Medical Officer for the Farcet District of the Peterborough Union. S. B. Stedman, M.R.C.S., L.R.C.P.Lond., appointed Medical Officer for the Swinhope District of the Caistor Union.

### THE NURSE'S REPORT BOOK.

Foolscap 4to., Black cover, **6d.** net. By Post 8d.

**Compiled by K. H. (Cert.)**

H. J. GLAISHER, 57, Wigmore Street, Cavendish Square, W.

